# The Early 2020 COVID-19 Outbreak in China and Subsequent Flourishing: Medium-Term Effects and Intervening Mechanisms

**DOI:** 10.1177/21568693221131819

**Published:** 2022-11-01

**Authors:** Yue Qian, Wen Fan

**Affiliations:** 1 The University of British Columbia, Vancouver, BC, Canada; 2 Boston College, Chestnut Hill, MA, USA

**Keywords:** mental health, psychosocial resources, economic strains, stress process, life course, COVID-19

## Abstract

In early 2020, a COVID-19 outbreak occurred in Hubei Province of China. Exploiting the geographic concentration of China’s COVID-19 cases in Hubei (the initial epicenter), we compare Hubei and non-Hubei residents to examine the medium-term effect of exposure to the COVID-19 outbreak on mental well-being. We examine flourishing—a comprehensive assessment of well-being that is not merely the absence of mental illness—and investigate a broad set of psychosocial and economic mediators that may link initial outbreak exposure to subsequent flourishing. We use ordinary least squares regression models to analyze national panel data collected in early 2020 and late 2021 (*N* = 3,169). Results show that flourishing scores remain lower for Hubei than non-Hubei residents almost two years following the early 2020 COVID-19 outbreak. Mediation analysis reveals that Hubei residents’ lower incidences of job promotion and lower sense of control are the two most important mediators accounting for their lower flourishing relative to non-Hubei residents. Combined, this study provides the first evidence of the medium-term psychological vulnerability borne by individuals who lived in the initial epicenter of the COVID-19 pandemic. Findings on the intervening mechanisms shed light on the policy initiatives needed for post-pandemic mental well-being recovery in China and other countries.

A growing body of research has documented the alarming implications of the COVID-19 pandemic for individuals’ mental well-being (see World Health Organization 2022 for a review). A systematic meta-analysis reveals that the global prevalence of anxiety and depression increased by a massive 25 percent in the first year of the pandemic (Santomauro et al. 2021). These studies have provided important evidence on the mental health toll of the pandemic, but they focused predominantly on the period immediately following the COVID-19 lockdowns or when the infection rates remained high (Montazer et al. 2022; Ran et al. 2020). In comparison, little is known about what happens when infections have subsided. Does previous exposure to an epidemic outbreak persevere such that its psychological impacts are palpable even years following the event? Understanding this question has become all the more important as the pandemic begins to wane and people’s lives gradually return to normal.

Extending the growing literature on mental health in the pandemic, we examine, for the first time, the medium-term effect of exposure to the COVID-19 outbreak on an important yet less-studied domain of well-being: flourishing. Flourishing integrates the emotional, social, and psychological dimensions of well-being to provide a more holistic view of mental health that enables individuals to live a better, more productive, and healthier life (Keyes 2002; Magyar and Keyes 2019). We leverage China as a strategic research site, given its unique timeline and geographic distribution of COVID-19. In late January 2020, a COVID-19 outbreak first occurred in Wuhan, the capital of Hubei Province. Given China’s stringent containment policies, COVID-19 cases were concentrated in Hubei with much fewer cases outside that region throughout the outbreak. By April 1, 2020, for example, over 80 percent of the cumulative COVID-19 cases in China (67,802 out of 81,589) were from Hubei (National Health Commission 2020). After over two months of strict lockdowns in Hubei, the mandate was lifted in April 2020 (State Council Information Office 2020). By comparison, other parts of China implemented much shorter lockdowns and began to reopen in early February of 2020 (Liu et al. 2021; also see Figure 1). Therefore, while Hubei residents lived through a lockdown of unprecedented scale and duration at a time when little was known regarding the novel virus (Qian and Hanser 2021), non-Hubei residents did not have as strong a direct experience with the outbreak. This regional distinction resembles a natural experiment in that an exogenous shock resulted in considerably different degrees of exposure to the COVID-19 outbreak between Hubei and non-Hubei residents.

**Figure 1.**
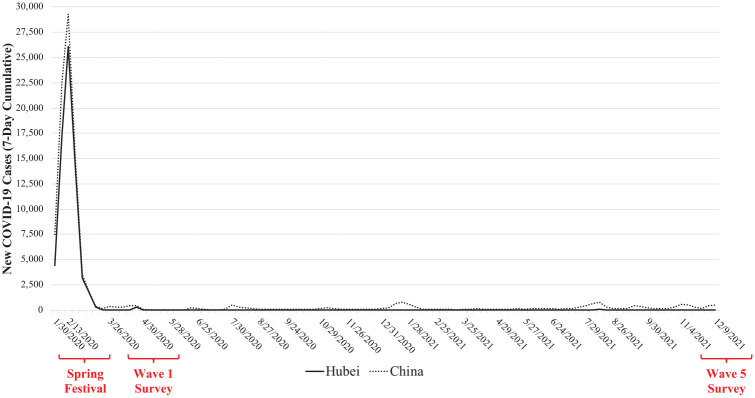
New COVID-19 cases (seven-day cumulative) in Hubei and China, January 24, 2020, to December 9, 2021. *Note*. The x-axis denotes the last day of each seven-day period. The 2020 Spring Festival was from January 24 to February 8, 2020. The Wave 1 survey was conducted from March 20 to April 28, 2020. The Wave 5 survey was conducted from October 28 to December 4, 2021. Data are from Johns Hopkins University’s COVID-19 Data Repository (Dong, Du, and Gardner 2020).

Would the disparate COVID-19 experiences lead to differential mental health outcomes for Hubei and non-Hubei people well past the initial exposure? To address this question, we draw on national, panel data collected from 3,169 Chinese respondents in March to April 2020 and October to December 2021. We have two research aims (see Figure 2 for our conceptual model). First, we examine differences in mental well-being between Hubei and non-Hubei residents almost two years following the early 2020 COVID-19 outbreak. Second, insofar as regional differences in mental well-being exist, we investigate a rich set of intervening psychosocial and economic pathways that may account for such differences.

**Figure 2.**
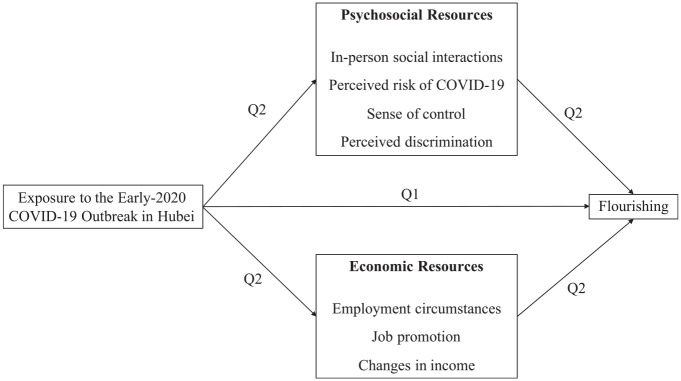
Conceptual model.

By addressing these research aims, this study contributes to the mental health literature in three critical ways. First, leveraging the COVID-19 outbreak as an exogenous shock, we deepen the understanding of the long arm of macro-level social change in shaping micro-level lived experiences. Building on the emerging studies that examined the immediate mental health effects of the COVID-19 pandemic (World Health Organization 2022), we begin to unravel the potentially long-lasting well-being impacts of prior exposure to the COVID-19 outbreak. Findings contribute to a life course understanding of the link between earlier-life adversity and subsequent health; they also facilitate predictions of population well-being post-pandemic, the first step toward effective policymaking in promoting human flourishing.

Second, moving beyond the existing focus on single dimensions of mental health—especially on mental disorders such as depression or anxiety—we examine flourishing, a multidimensional construct of mental health that integrates emotional, social, and psychological well-being (Keyes 2002). Flourishing captures individuals’ complete mental health such that flourishing individuals feel good about life *and* function well in life (Magyar and Keyes 2019). This shift of focus is important given that mental health is more than the absence of mental illness and that the prevention and treatment of mental illnesses will not necessarily lead to mentally healthy individuals (Magyar and Keyes 2019). Flourishing has also gained wide recognition in the popular press. A *New York Times* article, for example, posited flourishing to be a thriving state that individuals should aim for post-pandemic (Blum 2021). Our focus on flourishing engages with these arguments directly and contributes to a holistic understanding of mental health in the aftermath of social crises.

Third, in addition to examining the overall effect of COVID-19 on subsequent flourishing, we begin to unpack the black box. Drawing on stress process theory (Pearlin et al. 1981; Pearlin et al. 2005), we investigate an extensive set of mechanisms, including not only psychosocial factors such as a sense of control and in-person interaction but also important indicators of economic resources such as employment and income. These mediating factors help identify pathways that are especially pivotal in shaping human flourishing, thereby offering a much-needed empirical foundation for the development of policies that promote well-being recovery post-pandemic.

## Theoretical Frameworks

Our research is guided by the life course perspective and stress process theory (Elder 1999; Elder, Johnson, and Crosnoe 2003; Pearlin et al. 1981; Pearlin et al. 2005). The life course perspective emphasizes the connection between macro-level social change and micro-level lived experiences. In his classic book *Children of the Great Depression*, Glen H. Elder (1999) showed that early life exposure to the Great Depression had long-lasting effects on the life course of individuals who grew up during that era, evidenced by their distinct work trajectories and family lives compared with those from adjacent cohorts.

Time figures prominently in the life course tradition of linking the macro and the micro. Depending on the time frame examined, the same event could have positive, negative, or neutral effects on the life chances of affected individuals (Elder et al. 2003). Despite the generally negative, long-lasting effects found among large-scale events such as the Great Depression (Elder 1999) or the Great Recession (Burgard and Kalousova 2015), a repeated theme in life course studies is also one of resiliency and adaptation. Even in the face of extremely stressful events, humans gradually adapt after a period of struggle and volatility, exhibiting a high level of resiliency (Schoon 2006). Therefore, it remains an empirical question whether the negative mental well-being effect of living through the early 2020 COVID-19 outbreak persists or dissipates over time.

To understand the dynamics in the link between exposure to the COVID-19 outbreak and subsequent mental health, stress process theory calls for an evaluation of intervening processes (Pearlin et al. 2005). According to the notion of stress proliferation, people exposed to a serious adversity (primary stressor) could face a greater risk of later exposure to additional adversities (secondary stressors) that compound the negative impacts on mental well-being (Pearlin et al. 2005). As we discuss below, the COVID-19 outbreak may well have made a lasting imprint on mental health through triggering a series of secondary stressors. Although it is beyond the scope of this study to examine all possible intermediate factors, we focus on two broad sets of resources, namely, psychosocial and economic resources, as intervening mechanisms. This focus follows the stress process tradition—for example, the classic article by L. I. Pearlin and his colleagues (1981) examines economic strains and eroding positive concepts of self (self-esteem and mastery) as mechanisms linking involuntary job disruptions and depression. Pandemic-era research also shows considerable impacts on the economic and psychosocial resources of individuals who have been most adversely affected by the pandemic (Bierman and Schieman 2020; Bierman, Upenieks, Glavin, and Schieman 2021; Fan, Qian, and Jin 2021; Kovacs et al. 2021; Qian and Fan 2020; Tessler, Choi, and Kao 2020). It is therefore logical to conceptualize deteriorating economic and psychosocial resources as secondary stressors that mediate the relationship between exposure to the COVID-19 outbreak and subsequent flourishing.

## Regional Differences in China’s COVID-19 Containment Policies

To halt the fast-growing epidemic in January 2020, China implemented social distancing measures, but different control strategies were implemented inside and outside Hubei to strike a balance between epidemic control and economic development (Ke and Hsiao 2021; Liu et al. 2021). Wuhan, the capital of Hubei, went through a stringent, suddenly imposed lockdown starting on January 23, 2020, which included closing businesses, suspending public transportation, banning the use of private vehicles, and restricting individual movement (Qian and Hanser 2021). Other parts of Hubei were similarly recognized as key areas of epidemic growth and as such, they also implemented strict COVID-19 control measures (State Council Information Office 2020). Not until April 2020 were the strict quarantine requirements lifted for Hubei residents (Ke and Hsiao 2021). By comparison, although other provinces enacted social distancing measures soon after Hubei lockdowns, residents were primarily asked to stay vigilant against potential inbound transmissions, with daily lives beginning to return to normal in early February 2020 (Liu et al. 2021). In short, the COVID-19 containment measures were more restrictive and of a larger scale and longer duration in Hubei Province than in other parts of China.

## Exposure to the COVID-19 Outbreak and Subsequent Flourishing

Given the sudden nature and unprecedented scope of the early 2020 COVID-19 lockdowns in Hubei (Qian and Hanser 2021), the psychological trauma may have been long-lived for those who experienced the crisis firsthand. Previous studies have found long-term mental health impacts of large-scale social crises. For example, a study of the 1986 Chernobyl catastrophe shows that, even 20 years after this nuclear disaster, Ukrainians who received subclinical radiation doses exhibited poorer subjective well-being, higher depression rates, and lower subjective survival probabilities; they also relied more on governmental transfers as a source of subsistence (Danzer and Danzer 2016). A recent study of Hurricane Katrina survivors examines a sample of low-income mothers and reports that although post-traumatic stress symptoms declined over time, one in six still had symptoms indicative of probable post-traumatic stress disorder (PTSD) 12 years after the traumatic event (Raker et al. 2019). Similarly, a study of the Ebola epidemic finds that, after over a year of outbreak response, symptoms of PTSD and anxiety-depression remained common in the general population of Sierra Leone (an affected country), and these symptoms were even more likely to occur among individuals perceiving Ebola as an ongoing threat (Jalloh et al. 2018). In the Chinese setting, the SARS outbreak in the early 2000s was the most recent epidemic before COVID-19. Although little research has examined the SARS aftermath in the general population, a study of SARS survivors in Hong Kong shows that 25.6 percent had PTSD and 15.6 percent had depressive disorders 30 months after their infection—a pattern dubbed a “mental health catastrophe” (Mak et al. 2009). Given these findings, we expect that:

**Hypothesis 1:** Hubei residents have lower flourishing scores than non-Hubei residents almost two years following the early 2020 COVID-19 outbreak.

## Mechanisms Linking Exposure to the COVID-19 Outbreak and Subsequent Flourishing

Our research also aims to uncover the mechanisms through which exposure to the COVID-19 outbreak affects subsequent flourishing. Existing studies have shown that disasters and large-scale social change could set in motion a chain of intervening circumstances and events, which in turn lead to emergent or heightened post-crisis mental health problems (Davidson and McFarlane 2006; Fan 2016; Raker et al. 2019). Following the seminal article on stress process theory (Pearlin et al. 1981) and the growing studies on pandemic impacts that we review below, we expect psychosocial and economic resources to play a critical role in accounting for the potential difference in flourishing between Hubei and non-Hubei residents.

### Psychosocial Mediators

First, we expect the primary stressor, the COVID-19 outbreak, to result in a set of secondary stressors that are psychosocial in nature. Specifically, exposure to the early 2020 COVID-19 outbreak may lead Hubei residents to differ from their non-Hubei counterparts in the frequency of in-person interactions, perceived risk of COVID-19, sense of control, and perceived discrimination. In turn, these psychosocial stressors may lead to different levels of flourishing between Hubei and non-Hubei residents well after the COVID-19 outbreak.

Disasters have long been shown to disrupt social ties with extended family, close confidants, friends, and community networks, which are often sources of instrumental and emotional support (Bierman, Upenieks, and Schieman 2021; Kaniasty 2020). The infectious nature of COVID-19 may have led to longer term changes in behavioral patterns such as avoidance of in-person social interactions; the resulting disrupted access to social support networks may exacerbate the negative effects of a social crisis on mental well-being. U.S. research provides supportive evidence, showing significant decreases in network density and the size of extended acquaintance networks following a period of profound social isolation in June 2020 compared with June 2019 (Kovacs et al. 2021). Likewise, an increased sense of social isolation was observed among working Canadians between September 2019 and mid-March 2020 (when social distancing measures were enacted in Canada), which in turn predicted a rise in psychological distress (Bierman and Schieman 2020). Less is known, however, about whether this short-term deviation from the prepandemic pattern may have sustained into a longer term trend. We address this issue by examining Hubei residents’ frequency of in-person social interactions almost two years after the lockdown period, relative to that of non-Hubei residents with no lockdown experience.

Compared with non-Hubei residents, Hubei residents may also have greater concerns about the occurrence of another COVID-19 outbreak or the likelihood of themselves being infected with COVID-19. Although research is lacking on this topic in the context of the COVID-19 pandemic, the collective memory of living through a profound social change has been shown to have long-lasting effects. For example, individuals who grew up during the Great Depression are more likely to exhibit habits such as industry and thrift, possibly due to concerns about another depression (Elder 1999). Similarly, examining macroeconomic shocks during the 1960–2007 period, U. Malmendier and S. Nagel (2011) found that individuals who have experienced low stock market returns report lower willingness to take financial risks and are more pessimistic about future stock returns. Accordingly, the experience of living through the COVID-19 outbreak in early 2020 may have translated into a higher perceived likelihood of another outbreak. Similarly, given their direct exposure to the early 2020 outbreak, Hubei people may worry more about being infected with COVID-19 and thus exhibit lower flourishing, compared with non-Hubei people.

Sense of control—individuals’ perceived ability to control the exigencies that may confront them (Mirowsky and Ross 2003; Pearlin et al. 1981)—is another psychosocial mediator that we theorize to explain Hubei people’s potentially lower flourishing. Sense of control has been a prominent concept in mental health studies and is among the strongest predictors of mental well-being (Mirowsky and Ross 2003; Pearlin and Bierman 2013). The early 2020 COVID-19 outbreak in Hubei and the associated massive lockdowns were both exogenous shocks and were described by Hubei residents as “unexpected,” “inconceivable,” and “unprecedented” (Qian and Hanser 2021). Experiencing such a traumatic event firsthand—something far beyond individual control—may give rise to a sense of powerlessness and undermine one’s sense of control. We examine whether the potentially reduced sense of control partially explains the relationship between exposure to the COVID-19 outbreak and subsequent flourishing.

The last psychosocial mediator we consider is perceived discrimination, an important concept in mental health studies (Krieger 2000). Discrimination delivers the message that the targeted individuals are unworthy and dangerous, which threatens individuals’ needs for acceptance and inclusion, thereby undermining mental well-being (Goffman 1963; Link and Phelan 2001). Consistent with these arguments, perceived discrimination represents a dire psychosocial stressor that diminishes mental health (Kessler, Mickelson, and Williams 1999; Krieger 2000; Williams and Mohammed 2013). During the pandemic, discrimination against Chinese and Asians in general surged across the globe (Tessler et al. 2020). Similar dynamics surfaced in China, where Hubei people were stigmatized as “others” and blamed for producing and spreading the coronavirus; as a result, they perceived greater discrimination than non-Hubei people during and immediately following the early 2020 COVID-19 lockdowns (Fan et al. 2021). Left unknown, however, is whether perceived discrimination remains high among Hubei residents well after the outbreak. If this is the case, perceived discrimination may partly explain Hubei residents’ lower flourishing than non-Hubei residents, given the well-established link between perceived discrimination and psychological distress (Fan et al. 2021; Kessler et al. 1999; Williams and Mohammed 2013). Combined, we hypothesize that:

**Hypothesis 2:** Hubei residents’ lower flourishing than non-Hubei residents almost two years following the early 2020 COVID-19 outbreak is partly explained by Hubei residents’ (a) less frequent in-person interaction, (b) lower sense of control, (c) greater perceived likelihood of another COVID-19 outbreak, (d) greater perceived likelihood of themselves being infected with COVID-19, and (e) higher perceived discrimination.

### Economic Mediators

In addition to psychosocial factors, we theorize and empirically test a second set of economic factors that may mediate the relationship between exposure to the COVID-19 outbreak and subsequent flourishing. More than a public health crisis, the COVID-19 pandemic has sparked widespread economic downturns and job losses (World Bank 2020). In China, the economic impact of the early 2020 COVID-19 outbreak varied considerably by region. During the first quarter of 2020, the gross domestic product (GDP) of Hubei declined by 39.2 percent compared with a year prior (Hubei Provincial Statistics Bureau 2020), whereas the overall GDP decline in China was only 6.8 percent (National Bureau of Statistics 2020). Accordingly, we expect Hubei and non-Hubei residents to differ in their employment circumstances (including job insecurity and unemployment), job promotion prospects, and changes in income since the COVID-19 outbreak, which explain their disparity in flourishing.

From a social psychology perspective, insecurity represents a form of uncertainty for which individuals lack important information. Thus, job insecurity—the uncertainty about the continued existence of one’s job—often leaves one anxious and struggling, which deteriorates mental health (Alquist et al. 2020; Glavin and Schieman 2014). Unemployment similarly carries a high risk for well-being as it creates economic strain and disrupts individuals’ status, time structure, and interpersonal relationships (see Brand 2015 for a review). Empirical research has consistently demonstrated that both unemployed people (Brand 2015) and workers with high job insecurity (Burgard, Brand, and House 2009) show diminished mental well-being. There is also a large body of literature on the mental health threats induced by financial strain and economic hardship (e.g., Aneshensel 2009). In comparison, relatively little research exists on the well-being implications of job promotion, although this life event may well represent upward social mobility, signifying positive affirmation and thereby boosting mental health (Kidd 2008).

Since the onset of the COVID-19 pandemic, a growing body of evidence shows the economic suffering experienced by individuals (Bierman et al. 2021; Qian and Fan 2020; World Bank 2020). Declining economic growth and high unemployment rates imply that many workers lost their job, which likely heightened job insecurity for the employed as well (Lam, Fan, and Moen 2014; Wilson et al. 2020). Unemployment and job insecurity, given their well-documented mental health implications (Brand 2015; Burgard et al. 2009), may mediate the relationship between exposure to the COVID-19 outbreak and subsequent flourishing. In addition, the implementation of lockdowns lowered consumer spending and increased macroeconomic uncertainty (Coibion, Gorodnichenko, and Weber 2020), which may have diminished many workers’ opportunities for job promotion. In recession times, wage loss and income reduction are also common experiences, especially among individuals living in the hardest-hit regions. Recent research on China indeed shows that people who lived in Hubei during the early 2020 COVID-19 outbreak were more likely than non-Hubei residents to report income losses when surveyed between late March and late April in 2020 (Qian and Fan 2020).

What remains unclear however—and the focus of our research—is whether the pandemic-driven economic suffering represents temporary fluctuations that would disappear over time or, alternatively, leave an indelible mark even years after the initial outbreak. To the extent that economic recovery from recession takes time, we expect the economic toll disproportionately borne by Hubei people to persist over time, which partly explains their lower flourishing relative to non-Hubei residents almost two years later. Therefore, we hypothesize that:

**Hypothesis 3:** Hubei residents’ lower flourishing than non-Hubei residents almost two years following the COVID-19 outbreak is partly explained by Hubei residents’ (a) greater risk of unemployment and job insecurity, (b) greater likelihood of income reduction, and (c) diminished chances of job promotion.

## Method

### Data

In March to April 2020, we conducted an online survey in Mainland China through a professional survey firm. We oversampled residents in Hubei (the initial epicenter). To ensure sample diversity, we set quotas based on gender, age, and education within each stratum (Hubei, non-Hubei region). Although the survey was conducted online, respondents were recruited through various online and offline channels. To ensure data quality, we implemented protection mechanisms against bots and duplicate completions; we also included attention check questions and additional verification strategies (e.g., consistency between reported age group and birth year). The final sample of the Wave 1 survey contained 9,012 respondents, including 5,018 adults who lived in Hubei and 3,994 adults who lived in non-Hubei regions during the 2020 Chinese Spring Festival (January 24–February 8, 2020). The 2020 Spring Festival coincided with the period when COVID-19 cases were surging, especially in Hubei (see Figure 1). Respondents were followed up four times, in June–July of 2020 (Wave 2), November–December of 2020 (Wave 3), April–May of 2021 (Wave 4), and October–December of 2021 (Wave 5).

This study focuses on the 4,880 respondents who participated in Wave 5, the only wave that asked questions on flourishing. Of these 4,880 respondents, we first limit our analysis to 3,409 respondents of prime working age (25–54 years at Wave 1) given that economic and work circumstances are one set of mediators we focus on. We then restrict our sample to 3,179 (93 percent) respondents who resided in the same region (Hubei or non-Hubei) when surveyed in waves 1 and 5 to avoid confounding the mental health effect of exposure to the initial outbreak and that of subsequent moves in or out of Hubei. Last, excluding 10 respondents (0.3 percent) with missing data on the variables used yields an analytic sample of 3,169 respondents.

### Dependent and Independent Variables

All variables used in our analysis were measured at Wave 5 unless otherwise specified. *Flourishing* is our dependent variable, assessed through the Mental Health Continuum–Short Form (MHC-SF) (Keyes 2002). The MHC-SF contains 14 positively worded items that ask about respondents’ emotional well-being (three items), psychological well-being (six items), and social well-being (five items) during the past month. Each item is rated on a six-point scale: never, once or twice, about once a week, two or three times a week, almost every day, and every day. The MHC-SF has been translated and validated for use in Chinese adults (Yin and He 2012), which we follow in our survey. Because previous research has cautioned against using diagnostic categories to assess mental health (Mirowsky and Ross 2002), we follow recent research to measure flourishing as a continuous variable (Louie et al. 2021; Upenieks and Schieman, forthcoming). Flourishing scores are the sum of the 14 items (α = .96), ranging from 0 to 70.

*Hubei* is our independent variable, measuring respondents’ residence in either *Hubei* (=1) or *non-Hubei* (=0) region during the early 2020 COVID-19 outbreak (and Wave 5—recall that we limit our sample to those who resided in the same region when surveyed in waves 1 and 5).

### Mediators

#### Frequency of in-person socializing with friends

Respondents were asked how often they socialized with their friends in person during the past month, with response options of “never” (=0), “several times a month or less” (=1), and “several times a week” or “every day” (=2). We combined the last two options because only 23 respondents reported “every day.”

#### Likelihood of a COVID-19 outbreak

Respondents were asked: “Thinking about this winter, how likely do you think there will be a COVID-19 outbreak in the area you live in?” Response options included “not at all likely” (=0), “not too likely” (=1), “fairly likely” (=2), and “very likely” (=3).

#### Likelihood of COVID-19 infection

Respondents were asked: “If the COVID-19 pandemic continues, how likely do you think it is that you will be infected with COVID-19?” We recode the responses into a continuous variable, with 0 indicating “not at all likely,” 1 indicating “not too likely,” and 2 indicating “fairly likely” or “very likely.” We combined the last two categories because only 35 respondents reported “very likely.”

#### Sense of control

Respondents were asked, during the past week, how often they felt: (1) When something bad is about to happen, it will always happen no matter how hard you try to prevent it; (2) I have control over the direction my life is taking. For each item, respondents chose “never” (=0), “rarely” (=1), “sometimes” (=2), “often” (=3), or “always” (=4). We reverse-code the first item and sum the two items so that higher values indicate a greater sense of control.

#### Perceived discrimination

We measure perceived discrimination through the question: “How often did you feel being discriminated against during the past week?” Frequency was reported as “never” (=0), “rarely” (=1), “sometimes” (=2), “often” (=3), or “always” (=4). We did not ask respondents to specify the type or basis of discrimination to minimize confirmation bias. In addition, our regression analysis controls for major sources of discrimination in the Chinese context (e.g., gender, age, education, and *hukou*); therefore, the estimate can be interpreted as the net association between region-based discrimination in the aftermath of COVID-19 and flourishing while adjusting for alternative sources of discrimination.

#### Employment and job insecurity

We first distinguish between respondents who had a paid job, unemployed respondents (without a job yet looking for employment), and respondents who were out of the labor force (neither employed nor unemployed). For respondents with a job, we further differentiate those who reported “not at all likely,” “not too likely,” “fairly likely,” and “very likely” to the question, “Thinking about the next three months, how likely do you think it is that you will lose your job or be laid off?” Combined, this six-category variable allows us to classify respondents based on their labor market attachment and perceived job precarity.

#### Job promotion

Respondents were asked whether they had experienced a job promotion since the onset of the COVID-19 outbreak (Wave 1) or since the previous survey wave (waves 2–5). We code respondents as 1 if they answered “yes” in any of their participating waves and 0 otherwise. The number of participating waves is unlikely to affect our estimate for job promotion for two reasons. First, our analytic sample has a high retention rate: 72 percent of the 3,169 respondents participated in all five waves of the survey and another 17 percent were surveyed in four out of the five waves, with only 7 and 3 percent having participated in three and two waves of the survey, respectively. Second, as described below, our regression models control for the total number of participating waves as a covariate.

#### Change in income compared with prepandemic

Respondents were asked, compared with before the COVID-19 outbreak, whether they had more, less, or the same amount of income when surveyed; there was a fourth option for those who did not have any income as of the survey. We create a three-category variable to distinguish respondents who experienced an increase, no change, or a decrease (including 85 respondents who had no income when surveyed) in current income relative to prepandemic.

### Control Variables

We control for a series of potential confounders. First, because the MHC-SF scale was asked only in Wave 5, we control for respondents’ *psychological distress during the peak of China’s COVID-19 outbreak* (i.e., the 2020 Spring Festival), which was measured in Wave 1 through the 10-item Center for Epidemiologic Studies Depression (CESD) Scale (Andresen et al. 1994). Each item was rated on a four-point scale, ranging from zero (*rarely or none of the time*) to three (*most or all of the time*). We reverse-code two positively worded items and calculate the sum of the 10 items. This measure ranges from 0 to 30 with higher values indicating greater psychological distress. Including this measure is important because it allows us to control for any difference in baseline mental health between Hubei and non-Hubei residents.

Second, we control for a set of social and demographic covariates: *female* (=1 and *male* = 0), *age*, *university education or above* (=1 and *otherwise* = 0), *marital status* (married, previously married, never married, and cohabiting), and *prepandemic self-rated health* (1 = *poor*, 2 = *fair*, 3 = *good*, 4 = *very good*, and 5 = *excellent*). We also control for *rural* hukou (=1 and urban *hukou* = 0), which indicates respondents’ type of household registration, an important basis for access to resources in China (Qian and Fan 2020). Four of the six variables—gender, education, prepandemic self-rated health, and *hukou*—were measured at Wave 1.

Third, we control for *COVID-19 infection status*, a variable that likely differs between Hubei and non-Hubei residents and one that relates to mental health (Fan et al. 2021). In Wave 1, respondents were asked whether they had ever had COVID-19 (including those who showed COVID-19 symptoms but had not received a confirmed diagnosis). In waves 2 to 5, a similar question was asked about whether respondents had ever been infected with COVID-19 since the previous wave. We code respondents’ COVID-19 infection status as “yes” if they reported infection in any of their participating waves and “no” if they did not report any infection history; a few respondents chose the option “prefer not to say” and we include them in a separate category. Last, we control for the *total number of survey waves* in which respondents participated because it might affect measures such as job promotion and COVID-19 infection status. Descriptive statistics for all variables are shown in Table 1.

**Table 1.** Descriptive Statistics for Variables Used in the Analysis (*N* = 3,169).

Variables	*M*/%	*SD*
Flourishing	42.04	15.69
Hubei	54.34%	
Frequency of in-person socializing with friends	1.08	0.52
Likelihood of a COVID-19 outbreak	1.25	0.77
Likelihood of COVID-19 infection	0.90	0.65
Sense of control	5.11	1.54
Perceived discrimination	0.81	0.82
Employment and job insecurity		
Unemployed	3.56%	
Not in the labor force	7.66%	
Not at all likely to lose job	26.93%	
Not too likely to lose job	40.81%	
Fairly likely to lose job	16.66%	
Very likely to lose job	4.38%	
Job promotion	16.90%	
Change in income compared with prepandemic		
Increase	29.80%	
No change	39.41%	
Decrease	30.79%	
Psychological distress during the peak of China’s COVID-19 outbreak	7.86	5.40
Female	52.08%	
Age	34.74	7.07
University education or above	50.09%	
Marital status		
Married	65.68%	
Previously married	3.68%	
Never married	26.75%	
Cohabiting	3.89%	
Prepandemic self-rated health	4.02	0.96
Rural *hukou*	33.22%	
COVID-19 infection status		
No	97.96%	
Yes	0.81%	
Prefer not to say	1.23%	
Total number of waves	4.58	0.76

*Note*. All statistics are weighted by inverse probability weights (i.e., the inverse of the probability of being included in Wave 5).

### Analytical Strategies

We use ordinary least squares (OLS) regression models to analyze flourishing scores. Our first model includes the independent variable (Hubei versus non-Hubei residents) and control variables. The second model adds the theorized psychosocial and economic mediators. We use the Karlson–Holm–Breen (KHB) method (Kohler, Karlson, and Holm 2011) to estimate the extent to which each mediator explains the disparity in flourishing between Hubei and non-Hubei residents. To address any potential bias induced by attrition from Wave 1 to Wave 5, we use inverse probability weighting (Seaman and White 2013). All analyses were performed in Stata 16.1.

## Results

### Descriptive Results

Given our key interest in the differences between Hubei and non-Hubei residents, in Table 2, we examine whether these two groups differ in our dependent variable and the psychosocial or economic mediators. Compared with non-Hubei respondents, Hubei respondents scored much lower on flourishing (39.28 vs. 45.32).

**Table 2.** Descriptive Statistics for the Dependent Variable and Mediators, Hubei vs. Non-Hubei Residents.

	*M*/%		*p* value of significance test for difference between Hubei and non-Hubei
Variables	Hubei	Non-Hubei	
Flourishing	39.28 (14.77)	45.32 (16.14)	.000
Frequency of in-person socializing with friends	1.09 (0.51)	1.06 (0.54)	.062
Likelihood of a COVID-19 outbreak	1.23 (0.69)	1.28 (0.85)	.051
Likelihood of COVID-19 infection	0.93 (0.62)	0.86 (0.68)	.002
Sense of control	4.95 (1.44)	5.30 (1.63)	.000
Perceived discrimination	0.88 (0.80)	0.72 (0.85)	.000
Employment and job insecurity			.000
Unemployed	4.99%	1.85%	
Not in the labor force	10.14%	4.70%	
Not at all likely to lose job	21.73%	33.11%	
Not too likely to lose job	38.04%	44.10%	
Fairly likely to lose job	19.69%	13.07%	
Very likely to lose job	5.40%	3.17%	
Job promotion	11.61%	23.21%	.000
Change in income compared with prepandemic			.002
Increase	31.50%	27.78%	
No change	36.69%	42.65%	
Decrease	31.82%	29.56%	

*Note*. Standard deviations are in parentheses. All statistics are weighted by inverse probability weights (i.e., the inverse of the probability of being included in Wave 5).

Table 2 also sheds light on the psychosocial and economic impacts of exposure to the early 2020 COVID-19 outbreak. Contrary to our expectations, by Wave 5, respondents in Hubei did not show greater withdrawal from face-to-face interactions; nor were they more concerned about another COVID-19 outbreak. If anything, relative to non-Hubei residents, Hubei residents socialized with their friends in person more frequently (1.09 vs. 1.06, *p* = .062), and they also considered a COVID-19 outbreak in the 2021 winter season less likely (1.23 vs. 1.28, *p* = .051). The regional differences in other psychosocial mediators are consistent with our expectations. By Wave 5 when almost two years had passed since the early 2020 COVID-19 outbreak, Hubei respondents still reported a greater likelihood of themselves being infected with COVID-19 should the pandemic continue (0.93 vs. 0.86); they also demonstrated a lower sense of control (4.95 vs. 5.30) and higher perceived discrimination (0.88 vs. 0.72), compared with non-Hubei respondents.

Furthermore, Table 2 shows evidence for the lingering economic and employment impact of the early 2020 COVID-19 outbreak on Hubei residents. By Wave 5, Hubei respondents were more likely than non-Hubei respondents to be unemployed (5 vs. 2 percent) or out of the labor force (10 vs. 5 percent); even when employed, Hubei respondents reported greater job insecurity. For example, 25 percent of Hubei respondents but only 16 percent of non-Hubei respondents felt “very likely” or “fairly likely” to lose their job. By contrast, the percentage of those who felt “not at all likely” to lose their job was lower among Hubei than non-Hubei respondents (22 vs. 33 percent). Differential employment-related experiences between Hubei and non-Hubei respondents were also manifested in job promotion. Since the onset of the early 2020 COVID-19 outbreak, 23 percent of non-Hubei respondents were promoted at work at least once, whereas the percentage was only 12 percent among Hubei respondents. Last, Hubei respondents appeared more likely than non-Hubei respondents to experience change in income: 32 percent of Hubei respondents reported an increase and another 32 percent reported a decrease in income compared with prepandemic, whereas the respective figures were 28 and 30 percent for non-Hubei respondents.

### OLS Regression Results

Table 3 presents OLS regression results. Model 1 indicates that holding the control variables constant, the flourishing score is 3.370 points lower for Hubei than non-Hubei residents (*p* < .001). Although not our focus, the control variables show that the flourishing score is higher for women, younger or older respondents (relative to those in their mid-30s), highly educated respondents, married respondents, respondents with no COVID-19 infection history (relative to those with undisclosed status), and respondents with better psychological or physical health at baseline (all *p* < .05).

**Table 3.** Ordinary Least Squares Regression Models Predicting Flourishing.

	Model 1		Model 2	
Variables	Coef.	*SE*	Coef.	*SE*
Hubei	−3.370***	(0.519)	−2.027***	(0.445)
Frequency of in-person socializing with friends			2.065***	(0.418)
Likelihood of a COVID-19 outbreak			−0.653*	(0.320)
Likelihood of COVID-19 infection			−1.430***	(0.398)
Sense of control			3.633***	(0.179)
Perceived discrimination			−3.265***	(0.318)
Employment and job insecurity (ref. = Not at all likely to lose job)				
Unemployed			−3.976**	(1.307)
Not in the labor force			−2.840**	(0.934)
Not too likely to lose job			−3.207***	(0.513)
Fairly likely to lose job			−5.166***	(0.767)
Very likely to lose job			−6.420***	(1.237)
Job promotion			3.871***	(0.553)
Change in income compared with prepandemic (ref. = Increase)				
No change			0.025	(0.520)
Decrease			−0.281	(0.551)
Psychological distress during the peak of China’s COVID-19 outbreak	−0.913***	(0.052)	−0.340***	(0.046)
Female	1.932***	(0.509)	1.864***	(0.424)
Age	−0.807*	(0.370)	−0.650*	(0.303)
Age squared	0.012*	(0.005)	0.009*	(0.004)
University education or above	1.723**	(0.542)	0.756	(0.446)
Marital status (ref. = Married)				
Previously married	−4.947***	(1.400)	−3.097**	(1.194)
Never married	−2.781***	(0.646)	−2.415***	(0.540)
Cohabiting	−5.068***	(1.415)	−4.225***	(1.103)
Prepandemic self-rated health	2.307***	(0.279)	0.952***	(0.237)
Rural *hukou*	−0.129	(0.573)	0.755	(0.472)
COVID-19 infection status (ref. = No)				
Yes	3.479	(2.436)	4.818*	(2.198)
Prefer not to say	−5.649*	(2.663)	−4.074*	(1.843)
Total number of waves	−0.228	(0.339)	−0.219	(0.293)
Constant	54.946***	(7.366)	39.706***	(6.216)
*R* ^2^	.211		.481	

*Note*. Analyses are weighted by inverse probability weights (i.e., the inverse of the probability of being included in Wave 5). coef. = coefficient; ref. = reference category; *SE* = standard error.

* *p* < .05. **p < .01. ***p < .001 (two-tailed tests).

Model 2 presents the results after mediators are included. Our main finding holds: flourishing scores are 2.027 points lower for Hubei than non-Hubei residents (*p* < .001). Meanwhile, most of the mediators are significant. Mediators that are positively associated with flourishing include: more frequent in-person socializing with friends, lower perceived likelihood of a COVID-19 outbreak or COVID-19 infection, a greater sense of control, lower perceived discrimination, being employed with lower job insecurity, and job promotion (all *p* < .05). The only mediator that does not predict flourishing is change in income relative to prepandemic.

### Results From Mediation Analysis

Table 2 reveals considerable differences between Hubei and non-Hubei residents in their flourishing scores as well as psychosocial and economic resources. Table 3 further shows that most of the psychosocial and economic mediators predict flourishing in a significant way. These results lead to the question of to what extent the mediators explain the flourishing disparity between Hubei and non-Hubei residents. In Table 4, we present the KHB results to answer this question.

**Table 4.** Results of Mediation Analysis Using the Karlson–Holm–Breen (KHB) Method.

	Coefficient/% explained
Panel A (Coefficient for Hubei)	
Reduced	−3.370***
Full	−2.027***
Difference	−1.343***
Panel B (% Explained)	
Total	39.85%
Frequency of in-person socializing with friends	−2.14%
Likelihood of a COVID-19 outbreak	−2.21%
Likelihood of COVID-19 infection	1.19%
Sense of control	12.63%
Perceived discrimination	5.98%
Employment and job insecurity (total, see note)	11.39%
Job promotion	12.98%
Change in income compared with prepandemic (total, see note)	0.03%

*Note*. For categorical mediators with more than two categories, the estimated contribution of each category depends on the choice of the reference group, but the total contribution of the categorical variable is unaffected by the reference group (Yun 2005). For two such mediators—employment and job insecurity as well as change in income compared with prepandemic—we therefore only report the total contribution.

*** *p* < .001 (two-tailed tests).

Panel A of Table 4 shows that without controlling for the mediators, the flourishing score would be 3.370 units lower for Hubei than non-Hubei residents; after controlling for the mediators, the difference becomes 2.027, meaning a reduction of 1.343 units in the coefficient from the reduced to the full model (*p* < .001). The psychosocial and economic mediators thus explain 40 percent (= 1.343/3.370) of the flourishing disparity between Hubei and non-Hubei residents.

Which mediators are the most instrumental in accounting for the flourishing disparity? Results in Panel B of Table 4 quantify the role of each mediator. We note a few key findings. First, job promotion and sense of control are the most important mediators, with each explaining about 13 percent of the flourishing disparity between Hubei and non-Hubei residents. Second, employment and job insecurity are other notable mediators; jointly, they explain 11 percent of Hubei residents’ lower flourishing relative to non-Hubei residents. Last, we note two suppression effects. Recall that, relative to their non-Hubei peers, Hubei residents had slightly more in-person interaction with their friends (*p* = .062); they also considered it less likely that a COVID-19 outbreak would occur in the 2021 winter season (*p* = .051). If it were not for these two protective factors, Hubei residents would have had an even lower flourishing score, evidenced by the negative values in Table 4 denoting the percentages explained by these two mediators (−2.14 and −2.21 percent, respectively).

## Discussion

The COVID-19 pandemic has created and even widened mental health disparities by gender, race/ethnicity, social class, parental status, and other social dimensions (Moen 2022; Montazer et al. 2022; Shim 2020). Building on the growing pandemic studies, we draw on panel data collected in China nearly two years apart to examine the effect of exposure to the early 2020 COVID-19 outbreak on subsequent flourishing. Leveraging the exogenous shock of the outbreak that led to considerably different degrees of exposure to COVID-19 by region, we compare residents in Hubei (the initial epicenter) with those living in non-Hubei regions.

We show that flourishing scores remain lower for Hubei than non-Hubei residents almost two years following the initial outbreak. Considering that our data collection started after the onset of COVID-19, it is theoretically possible that Hubei residents exhibited lower psychological well-being than non-Hubei residents even well before the outbreak. Secondary nationally representative data collected in 2018, however, indicate that Hubei and non-Hubei residents reported similar mental health before the outbreak.^1^ Therefore, the early 2020 COVID-19 outbreak was indeed an exogenous shock, a shock whose mental well-being impacts were still palpable for Hubei residents well after the initial outbreak.

It is worth noting that Hubei residents exhibited lower flourishing despite China’s extremely low COVID-19 cases following the early 2020 outbreak (until a recent outbreak in Shanghai, which occurred after our Wave 5 data collection). If left unaddressed, the regional gap in flourishing may translate into more severe consequences for population health and health inequalities in the long run (Louie et al. 2021). Moving beyond existing studies that focused largely on the immediate pandemic impacts (e.g., Bierman and Schieman 2020; Fan et al. 2021; Montazer et al. 2022; Ran et al. 2020; World Health Organization 2022), our study advances a life course understanding by illustrating how the COVID-19 outbreak—a macro-level disruptive event—shapes micro-level lived experiences and individual well-being years following the outbreak. Our study also extends a small body of literature on the longer term mental health impacts of previous infectious disease outbreaks that occurred in China, which tended to focus on survivors of the diseases (Mak et al. 2009) or health care professionals (McAlonan et al. 2007). Our finding indicates that mental health issues occur more broadly, not merely among individuals who have direct exposure to the virus, a point we return to below when discussing the clinical implications of this study.

We further show that several economic and psychosocial factors—job promotion, employment and job insecurity, and sense of control—play a major role in explaining the flourishing disparity between Hubei and non-Hubei residents. Although we focus on the COVID-19 pandemic, we argue that, in general, economic and psychosocial mechanisms act as key intervening pathways in the stress process (Pearlin et al. 1981; Pearlin et al. 2005), and are critical in understanding the medium- or long-term mental health impacts of other natural or social disasters. Building on existing studies on pandemic-induced job loss, unemployment, and concerns about job insecurity (Wilson et al. 2020; World Bank 2020), we show that regional variations in these adversities—due to differential exposure to the COVID-19 outbreak and its associated containment measures—contribute to mental health disparity between Hubei and non-Hubei residents. In addition, our study makes a unique contribution to studies of mental health by highlighting a less-examined labor market process that nevertheless has profound impacts on individuals’ well-being—promotion at work. The diminished opportunities for job promotion turn out to be the most important mediator in explaining Hubei residents’ lower flourishing. Thus, future studies and intervention policies on mental well-being recovery need to consider not only job stability but also workers’ prospects of moving up the career ladder.

Our study also contributes by advancing a structural understanding of sense of control and assessing its role in linking social change and mental well-being. Despite a rich body of research that examined the role of sense of control in buffering the negative mental health impacts of disasters (for a study conducted during COVID-19, see Schnell and Krampe 2020), almost all of them treated sense of control as given. To the best of our knowledge, ours is the first to reveal the role of sense of control in explaining the disproportionate mental health risk borne by individuals living in the COVID-19 epicenter. Our findings demonstrate that, rather than being static, perceived control over personal lives can be directly altered by exposure to a disaster. To promote post-pandemic well-being recovery, this study highlights the urgent need for developing support policies to enhance sense of control among people who lived through the COVID-19 outbreak firsthand.

Results for other mediators provide suggestive evidence that residents in the hardest-hit region are not merely passive victims of disruptive events; rather, they exhibit high levels of resiliency. Despite a novel infectious disease outbreak and extremely stringent lockdowns (Qian and Hanser 2021), we find that by late 2021, Hubei and non-Hubei residents were comparable as to how often they socialized with friends in person and how concerned they were about the occurrence of a COVID-19 outbreak in their region. It appears that, at least with respect to face-to-face interactions and outlooks for future outbreaks, individuals gradually return to baseline levels over time, which has helped to protect their mental well-being from declining even further.

What are the clinical implications of our findings for other countries? Similar to what we show in China, in other parts of the world the mental health burdens can be disproportionately shouldered by individuals living in the hardest-hit regions with extended lockdowns (such as New York City, Italy; Day 2021). To promote flourishing, more resources need to be allocated to these regions. For example, residents in those places may receive regular clinical screening for mental health, which should last well after the COVID-19 outbreak begins to wane. Given that mental health conditions are still largely stigmatized, especially in low- and middle-income countries, appropriate mental health interventions designed for different population subgroups are essential to providing instrumental and psychological support. News and social media providing educational materials on mental health could also be useful for the general population.

We note a few limitations of this study. First, our survey was based on a non-random sample, which limits our ability to generalize. Highly educated people, for example, were overrepresented in our sample; this overrepresentation, however, occurred to a similar extent between Hubei and non-Hubei respondents (according to supplementary analyses that compared our sample with the nationally representative Chinese General Social Survey sample). Thus, our nonrepresentative sampling is unlikely to substantially affect the comparison between Hubei and non-Hubei respondents, the focus of this study. Second, given that questions on flourishing were only asked in one survey wave, we are unable to control for the corresponding baseline measure. We nevertheless include respondents’ psychological and physical health at baseline to capture changes in mental well-being to the extent possible. Third, although we include a comprehensive set of mediators, the psychosocial and economic factors we examine only account for 40 percent of the flourishing disparity between Hubei and non-Hubei residents. Future research is needed to theorize and empirically assess other mechanisms that explain the longer term mental health ramifications of COVID-19. Last, our mediators are mostly single-item measures; future research drawing on multi-item scales is necessary to increase measurement reliability. The measure of perceived discrimination, for example, could specify different sources of discrimination to better understand what type of discrimination was most well-being threatening in the aftermath of COVID-19.

Leveraging China’s early 2020 COVID-19 outbreak as a natural experiment, we examine an important yet understudied domain of mental well-being—flourishing—among Hubei and non-Hubei residents almost two years following the outbreak. We reveal persisting, negative mental health impacts of the COVID-19 outbreak and identify key economic and psychosocial mechanisms that account for Hubei residents’ mental health disadvantages—diminished opportunities for job promotion, lower sense of control, and reduced chances of being employed with high job security. Given the still ongoing pandemic, our study provides much-needed evidence on the sustained mental health adversity brought by COVID-19. To facilitate post-pandemic flourishing, our findings call for a continuation of policies aimed at promoting economic recovery and improving individuals’ sense of control, even well after COVID-19 cases have subsided.

## Notes

1. We examined the pre-outbreak difference in psychological distress between Hubei and non-Hubei residents by using nationally representative data from the China Family Panel Studies (CFPS) in 2018 (the most recent pre-outbreak data). The CFPS contained the eight-item CESD Scale, with each item rated on a four-point scale of 0–3 (Zhang and Zhao 2021). Averaging the eight items, we created a measure of psychological distress that ranged from 0 to 3. In 2018, the difference in psychological distress between Hubei and non-Hubei residents was small in magnitude (*difference*_Hubei–non-Hubei_ = −0.03) and nonsignificant (*p* = .321).
